# Comparison of dexmedetomidine vs. remifentanil combined with sevoflurane during radiofrequency ablation of hepatocellular carcinoma: a randomized controlled trial

**DOI:** 10.1186/s13063-018-3010-z

**Published:** 2019-01-08

**Authors:** Jingru Pan, Xianlong Li, Ye He, Chaojun Jian, Hui-xin Chen, Ziqing Hei, Shaoli Zhou

**Affiliations:** 1 Department of Anesthesiology, Third Affiliated Hospital, Sun Yat-sen University, Guangzhou, Guangdong 510630 People’s Republic of China; 2 Department of Anesthesiology, The University of Hong Kong-Shenzhen Hospital, Shenzhen, Guangdong People’s Republic of China

**Keywords:** Catheter ablation, Dexmedetomidine, Pain measurement

## Abstract

**Background:**

Remifentanil is widely used for ultrasound-guided percutaneous radiofrequency ablation (RFA) of small hepatocellular carcinoma (HCC). We determined whether dexmedetomidine could be an alternative to remifentanil for RFA of HCC under general anesthesia with sevoflurane.

**Methods:**

We prospectively randomized patients scheduled to undergo RFA for HCC to a dexmedetomidine (DEX) group or remifentanil (REMI) group (47 patients each). In the DEX group, a bolus infusion (0.4 μg kg^− 1^) was started 15 min before anesthesia induction and continued at 0.2 μg kg^− 1^ h^− 1^ until 10 min before the end of surgery. In the REMI group, 3 μg kg^− 1^ h^− 1^ of remifentanil was administered from 15 min before anesthesia induction to the end of the surgery. The primary endpoint was postoperative pain intensity. Secondary endpoints included analgesic requirement, postoperative liver function, patient comfort, and hemodynamic changes. Group allocation was concealed from patients and data analysts but not from anesthesiologists.

**Results:**

Postoperative pain intensity, analgesic consumption, comfort, liver function, and time to emergence and extubation did not differ between the two groups. Heart rate, but not mean arterial pressure, was significantly lower in the DEX group than in the REMI group, at 1 min after intubation and from 30 min after the start of the surgery until anesthesia recovery. Sevoflurane concentration and dosage were significantly lower in the DEX group than in the REMI group.

**Conclusion:**

During RFA for HCC, low-dose dexmedetomidine reduced the heart rate and need for inhalational anesthetics, without exacerbating postoperative discomfort or liver dysfunction. Although it did not exhibit outstanding advantages over remifentanil in terms of pain management, dexmedetomidine could be a safe alternative adjuvant for RFA under sevoflurane anesthesia.

**Trial registration:**

Chinese Clinical Trial Registry, ChiCTR-OPC-15006613. Registered on 16 June 2015.

**Electronic supplementary material:**

The online version of this article (10.1186/s13063-018-3010-z) contains supplementary material, which is available to authorized users.

## Background

Ultrasound-guided percutaneous radiofrequency ablation (RFA) is one of the most effective treatments for small hepatocellular carcinomas (HCCs) [1, 2]. RFA achieves complete ablation in 90–95% cases of small HCCs, with 5- and 10-year survival rates comparable to those after surgery [3–6]. Owing to its superior tumor control, high survival rates, minimally invasive nature, and ease of use, RFA has become the first-line treatment for small HCCs, especially in patients who are not eligible for surgical resection or liver transplantation [7, 8]. Percutaneous RFA can be performed under sedation, local anesthesia, or general anesthesia. However, some patients experience severe pain and anxiety during RFA under local anesthesia, which results in lower patient satisfaction and insufficient tumor ablation [9]. General anesthesia provides better pain control, better tolerance, and lower local recurrence rates [10].

Remifentanil, an ultra-short-acting μ-opioid-receptor agonist, has been demonstrated to be safe and reliable for RFA [11, 12]. As an adjuvant drug, remifentanil provides continuous analgesia and stable hemodynamics, but can cause cardiovascular side effects such as bradycardia, atrioventricular or sinoatrial block, and hypotension [13]. Dexmedetomidine is a highly selective α_2_-adrenoreceptor agonist with sedative, anxiolytic, and analgesic effects [14, 15]. It also exhibits neuroprotective properties [16], anti-inflammatory benefits [17], and protective effects on the myocardium [18], against ischemia or reperfusion in the brain [19], and against lung [20], kidney [21], and liver injuries [22, 23]. In patients undergoing percutaneous RFA of small HCCs under general anesthesia with sevoflurane, it is unclear whether dexmedetomidine exhibits outstanding advantages over remifentanil in terms of pain management, or if it could be an alternative to remifentanil.

Thus, the purpose of this prospective randomized study was to compare the effects of dexmedetomidine and remifentanil on postoperative pain intensity, analgesic requirements, liver function, and general comfort in patients undergoing RFA of HCCs under general anesthesia with sevoflurane.

## Methods

### Ethics

Ethical approval for this randomized prospective controlled study was provided by the ethics committee of the Third Affiliated Hospital of Sun Yat Sen University, Guangzhou, China, on 5 May 2015. This study was registered with the Chinese Clinical Trial Registry (ChiCTR-OPC-15006613, http://www.chictr.org.cn/edit.aspx?pid=11243&htm=4) on 16 June 2015. The checklist from the CONSORT 2010 Statement was used (Additional file 1).

### Patients and selection criteria

The study involved patients who were scheduled to undergo elective RFA for HCC under general anesthesia in our hospital between June 2015 and October 2015. The inclusion criteria were American Society of Anesthesiologists physical status classification I–II, Child–Pugh class A or B, age between 18 and 65 years, and a single tumor of size ≤5 cm or not more than three tumor nodules ≤3 cm. The exclusion criteria were: prior treatment for liver cancer (such as transarterial chemoembolization and liver resection); recent α_2_ agonist use; being allergic to any of the drugs used in this study; operation time <30 min or >3 h; history of serious impairment in respiratory, cardiovascular (heart block, myocardial ischemia, or uncontrolled high blood pressure), renal, or central nervous functions; long-term use of psychiatric or neurological drugs; and severe hearing disability. The patients were allowed to quit the study at any time.

After obtaining informed consent from all participants, trained staff used a computer-generated randomization code to randomize the patients into a remifentanil group (REMI group) or a dexmedetomidine group (DEX group) in a 1:1 ratio. For ethical reasons, patient safety, and drug dosing, the anesthesiologists in charge were not blinded to the study drugs, but group allocation was concealed from the patients and data analysts.

### Study design

Patients received no premedication. Heart rate (HR), peripheral arterial oxygen saturation (SpO_2_), non-invasive blood pressure, and bispectral index were monitored continuously (MP60, Philips, Boeblingen, Germany). In the DEX group, 200 μg dexmedetomidine diluted to a concentration of 4 μg mL^− 1^ was administered as a 0.4 μg kg^− 1^ bolus infusion 15 min before the induction of anesthesia and continued at a rate of 0.2 μg kg^− 1^ h^− 1^ until 10 min before the end of the surgery. In the REMI group, 1 mg remifentanil diluted to a concentration of 20 μg mL^− 1^ was administered continuously at a rate of 3 μg kg^− 1^ h^− 1^ from 15 min before the induction of anesthesia until the end of the surgery.

General anesthesia was induced with propofol (1.5 mg kg^− 1^), fentanyl (3.0 μg kg^− 1^), and cisatracurium (0.2 mg kg^− 1^). After tracheal intubation, anesthesia was maintained at a bispectral index of 45–55 with sevoflurane inhalation. At 5 min before the end of surgery, tropisetron was administered at a dose of 0.1 mg kg^− 1^ (maximum total dose, 5 mg). Sevoflurane was stopped at the end of the surgery (the end of the last RFA procedure).

On occasion, bolus doses of dopamine (2 mg) were administered to avoid hypotension (defined as a >30% decrease in mean arterial pressure [MAP] from the baseline value [before anesthesia induction]) [24, 25]. Atropine was administered at doses of 0.25 mg to avoid bradycardia (defined as HR < 50 beats per minute). These doses were repeated as necessary. Serious drug-related adverse events should be avoided, such as allergic reactions and refractory hemodynamic events (refractory hypotension and bradycardia were defined as hypotension or bradycardia that persisted for at least 10 min despite administering more than three doses of dopamine or atropine). If these events occurred intraoperatively, the anesthesiologist had immediately to terminate the drug infusion, take the necessary measures, and record the circumstances.

After the surgery, patients were transferred to a post-anesthesia care unit (PACU). Immediately when the patients awoke, they were extubated and questioned about their pain intensity and comfort. Pain intensity was assessed with a visual analog scale (VAS; 0–10 cm, handheld slide-rule type). If the VAS score was 3 or more, a bolus of 2 mg (body weight < 60 kg) or 3 mg (body weight > 60 kg) morphine [26–28] was administered intravenously. VAS scores were obtained every 5 min until pain relief, which was defined as a VAS score of <3. If the respiratory rate was <12 breaths/min, SpO_2_ was < 95%, or a serious adverse event (such as an allergic reaction, vomiting, or severe pruritus) related to morphine administration occurred, the morphine infusion was stopped. After the patients were transferred back to the ward, appropriate analgesics were administered if the patients complained of serious pain with VAS scores ≥3. Liver function was tested at least three times in the first three days after the surgery.

### Outcomes and data collection

The primary outcome (endpoint) was postoperative pain intensity, which was assessed by VAS scores. These scores were assessed every 5 min starting from the time of extubation in the PACU and were assessed daily during the first 3 postoperative days when the patients had returned to the ward. A trained investigator blinded to the group assignment performed the assessments for all patients.

Secondary outcomes (endpoints) included analgesic requirement, liver function, patient comfort, and hemodynamic changes. The intraoperative anesthetic requirement, which was the concentration of inhaled sevoflurane, was monitored every 15 min after the start of surgery, and the total dosage of sevoflurane was recorded. Postoperative analgesic administration, including that in the PACU and the ward, was also recorded. Preoperative liver function and the peak or nadir of liver-function data in the first three postoperative days were recorded, including aspartate aminotransferase (AST), alanine aminotransferase (ALT), albumin (ALB), total bilirubin (TBIL), prothrombin time (PT), and PT activity (PT%). Sedation–agitation scale (SAS) scores (Table 1) [29] were recorded every 5 min in the PACU. We also documented the time to emergence, which was defined as the interval between the end of the surgery and a response to a verbal command to open the eyes, which was assessed every 3 min, as well as the time to extubation. Patients’ general condition and comfort after the surgery were evaluated by assessing the rates of disorientation (patients were asked where they were), sore throats, hoarseness, headache, dizziness, discomfort, cold, nausea, vomiting, and intraoperative awareness. Hemodynamic data (HR and MAP) were monitored continuously and recorded at the following time points: before the administration of remifentanil or dexmedetomidine (baseline); 5, 10, and 15 min after their administration; at the time of intubation; 1, 2, and 5 min after intubation; at the start of the surgery; 15, 30, 45, and 60 min after the start of the surgery; when the patient was transferred to the PACU; 5 min after transfer to the PACU; at the time of extubation; and 1 and 5 min after extubation.

**Table 1:** Sedation–agitation scale (SAS)

Score	Term	Patients’ behavior
7	Dangerous agitation	Pulling at endotracheal tube, trying to remove catheters, climbing over bed rail, striking at staff, thrashing from side to side
6	Very agitated	Does not calm down despite frequent verbal reminders, requires physical restraints, bites endotracheal tube
5	Agitated	Anxious or mildly agitated, attempting to sit up, calms down with verbal instructions
4	Calm and cooperative	Calm, awakens easily, follows commands
3	Sedated	Difficult to arouse, awakens to verbal stimuli or gentle shaking but drifts off again, follows simple commands
2	Very sedated	Arouses to physical stimuli but does not communicate or follow commands, may move spontaneously
1	Unarousable	Minimal or no response to noxious stimuli, does not communicate or follow commands

### Statistical analysis

The primary outcome was the incidence of pain, defined as a VAS score ≥3. Our pre-experimental data indicated that this incidence would be 10% in the DEX group and 40% in the REMI group. The following formula [30] was used to determine the sample size: *n* = 15.7 / *h*^2^ where *h* = ∣Φ_1_ − Φ_2_∣, where Φ_1_ is the arcsine transformation for the DEX group and Φ_2_ is the arcsine transformation for the REMI group, assuming α = 0.05, β = 0.2, and a 20% dropout rate. Therefore, the study required 47 patients in each group.

Data were expressed as mean ± standard deviation, median and interquartile range, or proportions, and were analyzed using the SPSS v20.0 software package (SPSS, Chicago, IL, USA). Parametric data were analyzed using analysis of variance, and nonparametric data were analyzed using the Mann–Whitney *U*-test. MAP, HR, and sevoflurane concentration were evaluated using repeated-measures analysis of variance and the *t-*test. Time to emergence and time to extubation were compared using the Mann–Whitney *U*-test. Fisher’s exact test was used to analyze proportions. A two-sided *P* < 0.05 was considered statistically significant.

## Results

**Fig. 1:**
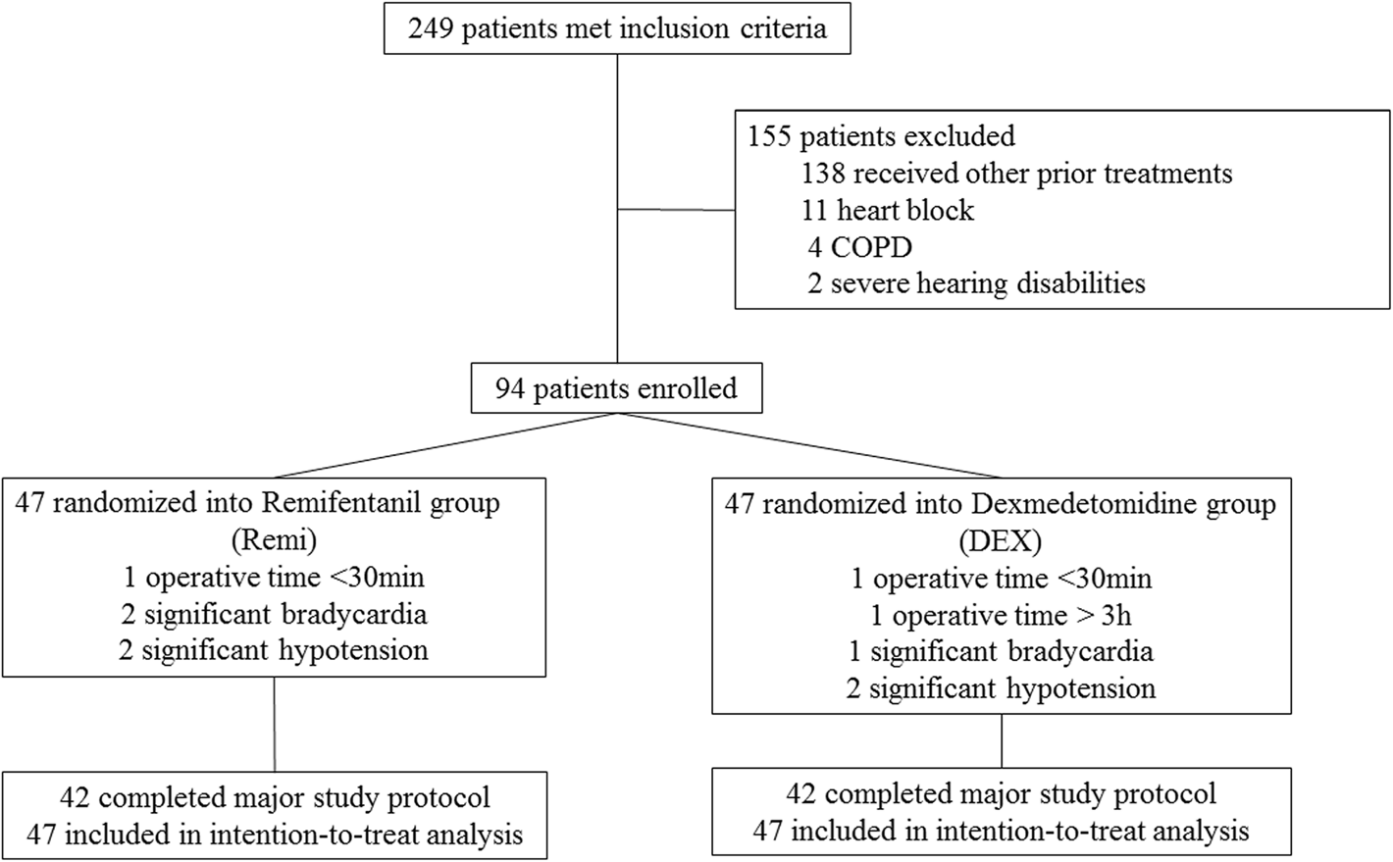
Patient enrollment and randomization. COPD chronic obstructive pulmonary disease

We screened 245 consecutive patients and enrolled 94 patients into this study. Five patients from each group either had an operative time outside our limits or experienced refractory intraoperative bradycardia or hypotension. Thus, finally, 42 patients in each group strictly completed the study protocol and in its entirety, but all 47 patients were included in the intention-to-treat analysis (Fig. 1 and Additional file 2). The baseline characteristics were well balanced between the two groups in terms of demographics and preoperative liver function (Table 2).

**Table 2:** Preoperative characteristics of the patients

Characteristic	Remifentanil	Dexmedetomidine
	*n* = 47 (50.00%)	*n* = 47 (50.00%)
Age (years)	49.57 ± 11.09	51.17 ± 10.15
Sex (male/female)	44/3	38/9
Body mass index (kg/m^2^)	22.73 ± 3.12	21.89 ± 2.65
Baseline AST (U/L)	37.60 ± 14.77	37.36 ± 20.12
Baseline ALT (U/L)	37.68 ± 22.20	35.55 ± 21.91
Baseline ALB (g/L)	40.20 ± 5.05	39.73 ± 4.17
Baseline TBIL (μmol/L)	20.15 ± 15.55	18.29 ± 12.56
Baseline PT (s)	14.46 ± 1.23	14.27 ± 1.59
Baseline PT% activity	85.34 ± 14.42	85.57 ± 14.49

Data are expressed as mean ± standard deviation, or numbers. None of the variables significantly differed between the two groups (*P* > 0.05)

*ALT* alanine aminotransferase, *AST* aspartate aminotransferase, *ALB* albumin, *TBIL* total bilirubin, *PT* prothrombin time

### Primary outcomes

VAS scores and the incidence of postoperative pain (VAS score ≥ 3) did not significantly differ between the two groups at any time point (*P* > 0.05; Table 3). The analgesic requirement did not significantly differ between the two groups within the time frame of the focus after the surgery (*P* > 0.05; Table 3). The incidence of postoperative pain at 48 h after surgery in the REMI group was significantly different from the incidence at the end of surgery (8.51% vs*.* 27.66%, *P* = 0.030). There were no significant differences at other time points in the same group (*P* > 0.05; Table 3).

**Table 3:** Incidence of postoperative pain, defined as a VAS score ≥ 3 and analgesic administration in the study groups

Parameter	Remifentanil (*n* = 47)	Dexmedetomidine (*n* = 47)	*P*
VAS score			
At the end of surgery	1 (0, 3)	0 (0, 2)	0.095
8 h after surgery	2 (0, 3)	1 (0, 3)	0.637
24 h after surgery	1 (0, 3)	0 (0, 2)	0.785
48 h after surgery	0 (0, 1)	0 (0, 2)	0.261
Patients with VAS score ≥ 3			
At the end of surgery	13 (27.66)	7 (14.89)	0.207
8 h after surgery	19 (40.43)	15 (31.91)	0.520
24 h after surgery	12 (25.53)	8 (17.02)	0.450
48 h after surgery	4 (8.51)*	9 (19.15)	0.231
Patients requiring analgesic administration			
Within 8 h after surgery	24 (51.06)	17 (36.17)	0.212
Within 72 h after surgery	30 (63.83)	24 (51.06)	0.297
Patients who required analgesics or had a VAS score ≥ 3 after transfer out of the PACU	26 (55.32)	22 (46.81)	0.536

Values expressed as median (25% percentile, 75% percentile), or number (percentages)

*PACU* post-anesthesia care unit, *VAS* visual analog scale

*P < 0.05 vs. patients with VAS score ≥ 3 at the end of surgery

### Secondary outcomes

Immediately after extubation, the general condition of the patients, in terms of disorientation, sore throat, hoarseness, headaches, dizziness, discomfort, cold, nausea, vomiting, and intraoperative awareness, was similar in both groups (*P* > 0.05; Table 4). Liver function was assessed on the first 3 postoperative days. The peak ALT, AST, and TBIL levels were slightly higher in the DEX group, while the nadir ALB and PT% activity and peak PT levels were slightly lower in the REMI group, but the differences were not statistically significant (*P* > 0.05; Table 5). All of the liver functions tested were significantly worse postoperatively than preoperatively (*P* < 0.05).

**Table 4:** General condition and comfort of patients after the surgery

	Remifentanil (*n* = 47)	Dexmedetomidine (*n* = 47)	*P*
Disorientation	5 (10.64)	5 (10.64)	1.000
Sore throat	8 (17.02)	9 (19.15)	1.000
Hoarseness	2 (4.26)	3 (6.38)	1.000
Headache	0 (0.00)	1 (2.13)	1.000
Dizziness	3 (6.38)	4 (8.51)	1.000
Uncomfortable	2 (4.26)	4 (8.51)	0.677
Cold	0 (0.00)	3 (6.38)	0.242
Nausea	2 (4.26)	7 (14.89)	0.158
Vomiting	0 (0.00)	0 (0.00)	–
Intraoperative awareness	0 (0.00)	0 (0.00)	–

Values expressed as number of patients with percentages in parentheses

**Table 5:** Postoperative laboratory data during the first 3 postoperative days

	Remifentanil (*n* = 47)	Dexmedetomidine (*n* = 47)	*P*
Peak AST (U/L)	293.15 ± 208.68	285.98 ± 131.67	0.843
Peak ALT (U/L)	240.48 ± 196.20	265.17 ± 207.06	0.557
Nadir ALB (g/L)	34.62 ± 5.44	32.92 ± 6.82	0.190
Peak TBIL (μmol/L)	42.32 ± 30.97	49.21 ± 55.05	0.468
Peak PT (s)	17.06 ± 6.26	16.23 ± 5.12	0.522
Nadir PT% activity	71.72 ± 12.68	73.54 ± 13.29	0.540

Values are expressed as mean ± standard deviation

*ALT* alanine aminotransferase, *AST* aspartate aminotransferase, *ALB* albumin, *TBIL* total bilirubin, *PT* prothrombin time

**Fig. 2:**
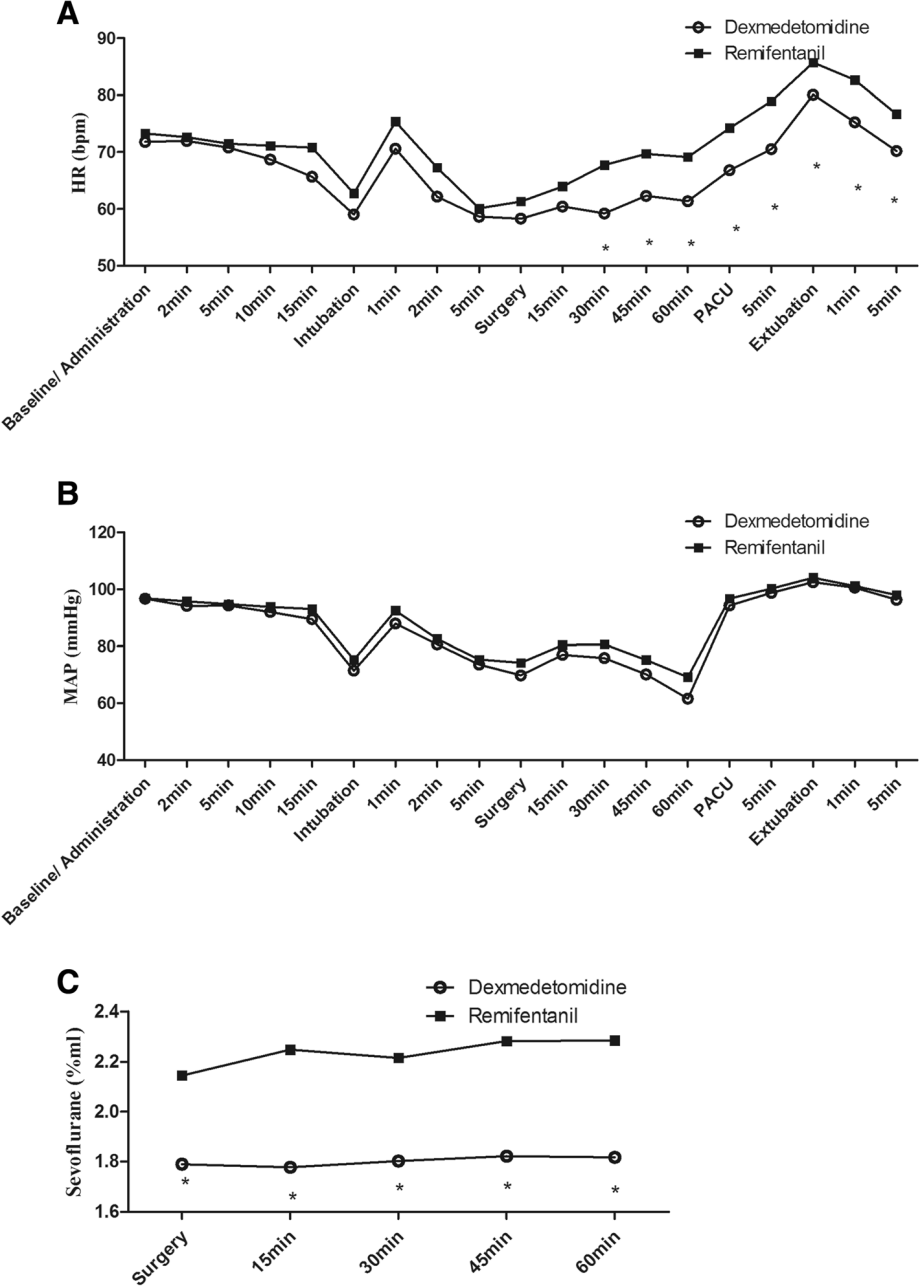
Hemodynamic changes during the observation period: **a** heart rate (HR), **b** mean arterial pressure (MAP), and **c** sevoflurane concentration during surgery. HR heart rate, MAP mean arterial pressure, PACU post-anesthesia care unit. *P < 0.05 vs*.* the REMI group

Repeated-measures analysis of variance revealed significant variation in HR and MAP over time within each group (Fig. 2a, b). HR was significantly lower in the DEX group than in the REMI group at 1 min after intubation (70.62 ± 12.93 vs*.* 75.38 ± 15.46, *P* = 0.018), 30 min after the start of surgery (59.21 ± 8.26 vs*.* 67.76 ± 12.90, *P* < 0.001), and during recovery (*P* < 0.005; Table 6). Hypotension (34.04% vs*.* 23.40%, *P* = 0.362) and bradycardia (34.04% vs*.* 40.43%, *P* = 0.670) (requiring treatment with dopamine or atropine) occurred in both groups, but their incidence did not differ between the two groups (Table 7). Refractory hypotension (4.26% vs*.* 4.26%, *P* = 1.000) and bradycardia (4.26% vs*.* 2.13%, *P* = 1.000) also occurred in both groups, but their incidences were not significantly different (Table 7).

**Table 6:** Intraoperative hemodynamic data

	Mean arterial blood pressure (mm Hg)			Heart rate (beats/min)		
	Remifentanil	Dexmedetomidine	*P*	Remifentanil	Dexmedetomidine	*P*
Baseline/ administration	96.83 ± 10.42	96.69 ± 9.70	0.943	73.28 ± 11.74	71.81 ± 13.41	0.574
2 min	95.74 ± 10.52	94.19 ± 10.77	0.481	72.64 ± 12.37	71.98 ± 13.65	0.807
5 min	94.79 ± 11.14	94.32 ± 9.91	0.830	71.47 ± 10.62	70.81 ± 13.44	0.792
10 min	93.81 ± 10.82	92.04 ± 10.45	0.423	71.11 ± 11.02	68.70 ± 13.89	0.355
15 min	93.04 ± 11.29	89.49 ± 11.53	0.135	70.83 ± 12.58	65.68 ± 12.75	0.052
Intubation	75.28 ± 11.00	71.53 ± 12.30	0.123	62.77 ± 9.54	59.06 ± 11.71	0.096
1 min	92.47 ± 15.35	87.94 ± 15.49	0.158	75.38 ± 15.46	70.62 ± 12.93	0.018
2 min	82.60 ± 11.31	80.70 ± 12.27	0.439	67.30 ± 13.06	62.36 ± 11.56	0.055
5 min	75.38 ± 10.10	73.66 ± 9.67	0.400	60.13 ± 10.84	58.64 ± 10.33	0.497
Surgery	74.21 ± 10.83	69.85 ± 11.20	0.058	61.32 ± 9.00	58.33 ± 9.50	0.122
15 min	80.43 ± 10.84	77.00 ± 16.01	0.299	63.98 ± 8.55	60.45 ± 9.73	0.071
30 min	80.68 ± 16.89	75.91 ± 17.40	0.183	67.76 ± 12.90	59.21 ± 8.26	< 0.001
45 min	75.26 ± 21.10	70.18 ± 25.82	0.306	69.71 ± 11.18	62.32 ± 8.44	0.002
60 min	69.22 ± 22.83	61.62 ± 24.67	0.139	69.15 ± 8.45	61.37 ± 9.61	0.001
PACU	96.72 ± 11.62	94.30 ± 11.98	0.322	74.24 ± 11.64	66.82 ± 13.01	0.005
5 min	100.26 ± 12.24	98.77 ± 12.24	0.577	78.93 ± 13.73	70.56 ± 14.42	0.006
Extubation	104.13 ± 13.85	102.47 ± 10.22	0.510	85.70 ± 14.11	80.09 ± 12.66	0.045
1 min	101.17 ± 12.63	100.57 ± 8.75	0.791	82.66 ± 13.08	75.23 ± 12.39	0.006
5 min	97.96 ± 9.41	96.30 ± 9.45	0.396	76.68 ± 12.07	70.21 ± 10.52	0.007

Values are expressed as mean ± standard deviation

*PACU* post-anesthesia care unit

**Table 7:** Number of patients with hypotension or bradycardia

	Remifentanil (*n* = 47)	Dexmedetomidine (*n* = 47)	*P*
Hypotension	11 (23.40)	16 (34.04)	0.362
Bradycardia	19 (40.43)	16 (34.04)	0.670
Refractory hypotension	2 (4.26)	2 (4.26)	1.000
Refractory bradycardia	1 (2.13)	2 (4.26)	1.000

Values expressed as numbers and percentages

At each measured time point during the surgery, the concentration of inhaled sevoflurane was significantly lower in the DEX group than in the REMI group (Fig. 2c). Anesthesia time did not significantly differ between the two groups, but there were significant differences in the total dosage of sevoflurane (22.77 ± 11.18 vs*.* 17.58 ± 11.22 mL, *P* = 0.017) and the sevoflurane dosage related to anesthesia time (16.41 ± 5.74 vs*.* 11.56 ± 5.20 mL h^− 1^, *P* < 0.001; Table 8). Delayed emergence was defined as a time to emergence of over 30 min. Although there were more patients with delayed emergence in the DEX group (12.77% vs*.* 4.26%), the difference was not statistically significant (*P* = 0.267; Table 9). Time to emergence, time to extubation, and the number of patients with a postoperative SAS score of ≥5 did not significantly differ between the two groups (Table 9).

**Table 8:** Dosage of sevoflurane

	Remifentanil (*n* = 47)	Dexmedetomidine (*n* = 47)	*P*
Anesthesia time (h)	1.37 ± 0.52	1.14 ± 0.64	0.767
Total dosage of sevoflurane (mL)	22.77 ± 11.18	17.58 ± 11.22	0.017
Sevoflurane related to anesthesia time (mL h^−1^)	16.41 ± 5.74	11.56 ± 5.20	< 0.001

**Table 9:** Emergence from anesthesia

	Remifentanil (*n* = 47)	Dexmedetomidine (*n* = 47)	*P*
Patients with delayed emergence	2 (4.26)	6 (12.77)	0.267
Time to emergence (min)*	14 (11, 18)	15 (10, 24)	0.066
Time to extubation (min)*	19 (15, 25)	19 (15, 29)	0.051
Patients with SAS ≥ 5 at extubation	2 (4.26)	6 (12.77)	0.267
Patients with SAS ≥ 5 at 1 min after extubation	0 (0.00)	2 (4.26)	0.495
Patients with SAS ≥ 5 at 5 min after extubation	0 (0.00)	1 (2.13)	1.000

Values expressed as numbers and percentages, or medians and interquartile ranges (25th percentile, 75th percentile)

*SAS* Sedation-Agitation scale

*Mann–Whitney *U*-test

## Discussion

Unlike other studies or our pre-experiment, this study showed that compared with remifentanil, dexmedetomidine did not significantly reduce postoperative pain or analgesic consumption in patients undergoing RFA for small HCCs. However, dexmedetomidine significantly reduced the demand for inhaled anesthetics and inhibited an increase in HR during extubation. It did not influence immediate postoperative patient comfort, exacerbate liver-function impairment during the first 3 postoperative days, or delay recovery from anesthesia.

Dexmedetomidine is a selective α_2_-adrenoreceptor agonist, with superior sedative, anxiolytic, and analgesic properties. Several studies have shown that dexmedetomidine decreases VAS scores, analgesic requirement, and opioid-related adverse events [31–33]. Dexmedetomidine exhibits a synergistic effect with the opioid system and might potentiate the analgesic effect of other analgesic drugs [34, 35]. In some reports, dexmedetomidine has exhibited superior efficacy in pain management compared to remifentanil during a PACU stay after general anesthesia [36, 37]. Dexmedetomidine also has opioid-sparing properties [38, 39]. This might partially explain why fewer patients in the DEX group had VAS scores of ≥3 in the present study. However, the difference was not statistically significant, and dexmedetomidine did not show any advantages over remifentanil in terms of VAS scores or analgesic consumption. The discrepancy between the above findings and those of our study might be attributable to the effect of local hyperthermia on the liver capsule, and peripheral nerves and vessels, and inadequate analgesia provided by the two study drugs. The analgesic effects of the drugs were dose dependent. In our study, dexmedetomidine and remifentanil were administered intraoperatively, and other preventive analgesic drugs were not used. Furthermore, although RFA is more rapid and less invasive than liver resection, unfortunately, 14.89–40.43% of patients complained of pain after RFA in this study.

Remifentanil can provide rapid recovery from anesthesia, but occasionally opioid-induced hyperalgesia can emerge after long-term infusion [40], though the evidence is conflicting [41]. The analgesic effect of remifentanil fades within 3–10 min. Immediately after extubation, 13 patients (27.66%) in the REMI group, who could be considered to be without any analgesia, experienced significant pain. At 48 h later, the pain caused by surgery was significantly reduced, and the proportion of patients complaining of pain decreased significantly (*P* = 0.030). The above difference might be due to the higher proportion at the end of surgery, and the rapid decline 48 h later.

Further analysis showed that the number of patients with pain peaked at 8 h after the surgery (40.43% and 31.91% in the REMI and DEX groups, respectively), and approximately half the patients in each group required analgesics or had a VAS score of ≥3 even after transferring out of the PACU. This suggests that in addition to the administration of analgesics at the end of the surgery, prophylactic analgesia may be required until the patient is transferred to the ward, especially in the first 8 h following surgery.

RFA is a safe treatment for HCC associated with mild liver dysfunction but has the potential to aggravate preexisting hepatic dysfunction [42]. Transient liver dysfunction after RFA is common. AST, ALT, and TBIL levels peak at 12–24 h after RFA [43]. Remifentanil is metabolized by nonspecific esterases present mainly in the blood and is considered to have no effect on liver function. In contrast, dexmedetomidine is metabolized by the liver, and one study has reported that high doses could induce oxidative stress in liver tissue [44]. However, several studies [22, 23, 45] have shown that dexmedetomidine has a protective effect against liver ischemia-reperfusion injury. In the present study, postoperative liver function was not significantly different between the two groups and the protective effect of dexmedetomidine was not observed. Possible reasons for this finding might be that the liver dysfunction caused by RFA was minimal and differed from ischemia-reperfusion injury. However, the precise reasons require further investigation.

Remifentanil provides hemodynamic stability and attenuates the stress response but is commonly associated with side effects, such as bradycardia (2–12%) and hypotension (6–30%), which are strongly dose dependent [46, 47]. Drops in HR, such as those observed in our study, are mainly caused by centrally mediated sympatholytic and/or vagotonic actions, whereas drops in blood pressure are mainly the result of direct vasodilation [48, 49]. Dexmedetomidine reduces catecholamine secretion, soothes the stress response to endotracheal intubation or extubation, and maintains hemodynamic stability during the intraoperative period, all of which are outstanding advantages [50, 51]. The cardiovascular effects of dexmedetomidine mainly result from peripheral and central α_2_-adrenoreceptor activation [52]. The reduction of stress and analgesic effects might play a key role. The causes of bradycardia might be central sympatholytic action, activation of the baroreceptor reflex, and enhanced vagal activity. This effect is expected to blunt changes in HR. Some researchers believe that significant hypotension caused by dexmedetomidine is usually observed only in patients with preexisting vasoconstriction or hypovolemia [53]. In one study, the incidence of hypotension requiring intervention was slightly higher in patients receiving high-dose dexmedetomidine than in those receiving lower doses [54]. In the present study, as all patients had some hepatic dysfunction, we selected low drug doses (0.4 μg kg^− 1^ bolus and maintenance with 0.2 μg kg^− 1^ h^− 1^), which we believed would have a lesser impact on hemodynamics, including hypotension and bradycardia [55–57]. Although hypotension and bradycardia still occurred in our study, HRs were stable during postoperative recovery in the DEX group.

Another important observation of our study was that dexmedetomidine, as previously reported [58], significantly reduced the demand for inhalational anesthetics, without affecting the time to emergence or extubation. Although one study [59] found that dexmedetomidine is associated with delayed recovery, several others [60–62] have reported that as an adjuvant to general anesthesia, dexmedetomidine results in more stable hemodynamics, better recovery, and easy extubation, without affecting recovery time. One possible reason for this difference is that inhaled anesthetics are rapidly discharged due to lower intraoperative demand, and the time to emergence and extubation might be influenced more by the anesthetic dose than by the administration of analgesics.

Our study has certain limitations. First, the specifics of the RFA procedures, including power output, time, and location, were not the same for each patient owing to differences in patient conditions and ethical requirements. A multicenter large-scale trial could resolve this problem. Second, for patient safety, the anesthesiologists in charge were not blinded to the drugs used in the surgeries, and this could lead to bias. Third, only 3 days of follow-up were performed, which might be inadequate to assess prognosis. Longer observation is necessary to obtain more complete and meaningful comparisons.

## Conclusion

In conclusion, the perioperative administration of low-dose dexmedetomidine to patients undergoing RFA reduced their HRs and the requirement for inhalational anesthetics, and did not exacerbate postoperative discomfort or liver dysfunction. Although dexmedetomidine did not reduce postoperative pain scores or exhibit an analgesic-sparing effect compared with remifentanil, it appeared to be a safe optional adjunct and it appeared that postoperative pain was strictly controlled in all RFA patients.

## Additional files

Additional file 1: CONSORT-Equity checklist. (DOCX 18 kb)

Additional file 2: CONSORT-Equity flow diagram. Intervention A denotes remifentanil and intervention B denotes dexmedetomidine. (DOC 70 kb)

## References

[1] Chen MS, Li JQ, Zheng Y, Guo RP, Liang HH, Zhang YQ, Lin XJ, Lau WY (2006). A prospective randomized trial comparing percutaneous local ablative therapy and partial hepatectomy for small hepatocellular carcinoma. Ann Surg.

[2] Lahat E, Eshkenazy R, Zendel A, Zakai BB, Maor M, Dreznik Y, Ariche A (2014). Complications after percutaneous ablation of liver tumors: a systematic review. Hepatob Surg Nutr.

[3] Lencioni R, Cioni D, Crocetti L, Franchini C, Pina CD, Lera J, Bartolozzi C (2005). Early-stage hepatocellular carcinoma in patients with cirrhosis: long-term results of percutaneous image-guided radiofrequency ablation. Radiology.

[4] Tateishi R, Shiina S, Teratani T, Obi S, Sato S, Koike Y, Fujishima T, Yoshida H, Kawabe T, Omata M (2005). Percutaneous radiofrequency ablation for hepatocellular carcinoma. An analysis of 1000 cases. Cancer.

[5] Yang W, Yan K, Goldberg SN, Ahmed M, Lee JC, Wu W, Zhang ZY, Wang S, Chen MH (2016). Ten-year survival of hepatocellular carcinoma patients undergoing radiofrequency ablation as a first-line treatment. World J Gastroenterol.

[6] N'Kontchou G, Mahamoudi A, Aout M, Ganne-Carrie N, Grando V, Coderc E, Vicaut E, Trinchet JC, Sellier N, Beaugrand M, Seror O (2009). Radiofrequency ablation of hepatocellular carcinoma: long-term results and prognostic factors in 235 Western patients with cirrhosis. Hepatology.

[7] Bruix J, Sherman M (2011). Management of hepatocellular carcinoma: an update. Hepatology.

[8] Livraghi T, Meloni F, Di Stasi M, Rolle E, Solbiati L, Tinelli C, Rossi S. Sustained complete response and complications rates after radiofrequency ablation of very early hepatocellular carcinoma in cirrhosis: Is resection still the treatment of choice? Hepatology 2008;47(1):82–89. Epub 2007/11/17. 10.1002/hep.21933 18008357

[9] Takasaki J, Arai K, Ando M, Nagahama T, Fukuda A, Ami K, Kurokawa T, Ganno H, Amagasa H, Watayou Y, Fujiya K, Nakamura M, Katagiri S, Yoneda G, Yamamoto M, Saito A (2012). Examination of the effect of anesthesia on radiofrequency ablation of hepatocellular carcinoma-a patient survey on anesthesia for radiofrequency ablation. Gan To Kagaku Ryoho Cancer Chemother.

[10] Lai R, Peng Z, Chen D, Wang X, Xing W, Zeng W, Chen M (2012). The effects of anesthetic technique on cancer recurrence in percutaneous radiofrequency ablation of small hepatocellular carcinoma. Anesth Analg.

[11] Yang LL, Ji JS, Wu W, Lei LP, Zhao ZW, Shao GL, Zheng JP (2013). Clinical observation of remifentanyl and propofol injection in total intravenous anesthesia for percutaneous radiofrequency ablation. Zhonghua Yi Xue Za Zhi.

[12] Li Y, Huang W, Long YH, Li W, Wang J, Chen MS, Xu MX (2007). Application of total intravenous anesthesia with remifentanyl and propofol to radiofrequency ablation. Ai Zheng.

[13] DeSouza G, Lewis MC, TerRiet MF (1997). Severe bradycardia after remifentanil. Anesthesiology.

[14] Bhana N, Goa KL, McClellan KJ (2000). Dexmedetomidine. Drugs.

[15] Hauber JA, Davis PJ, Bendel LP, Martyn SV, McCarthy DL, Evans MC, Cladis FP, Cunningham S, Lang RS, Campbell NF, Tuchman JB, Young MC (2015). Dexmedetomidine as a Rapid Bolus for Treatment and Prophylactic Prevention of Emergence Agitation in Anesthetized Children. Anesth Analg.

[16] Wang Y, Han R, Zuo Z (2016). Dexmedetomidine post-treatment induces neuroprotection via activation of extracellular signal-regulated kinase in rats with subarachnoid haemorrhage. Br J Anaesth.

[17] Liu Z, Wang Y, Ning Q, Zhang Y, Gong C, Zhao W, Jing G, Wang Q (2016). Dexmedetomidine attenuates inflammatory reaction in the lung tissues of septic mice by activating cholinergic anti-inflammatory pathway. Int Immunopharmacol.

[18] Xu L, Hu Z, Shen J, McQuillan PM (2014). Does dexmedetomidine have a cardiac protective effect during non-cardiac surgery? A randomised controlled trial. Clin Exp Pharmacol Physiol.

[19] Zeng X, Wang H, Xing X, Wang Q, Li W (2016). Dexmedetomidine Protects against Transient Global Cerebral Ischemia/Reperfusion Induced Oxidative Stress and Inflammation in Diabetic Rats. PLoS One.

[20] Jiang L, Li L, Shen J, Qi Z, Guo L (2014). Effect of dexmedetomidine on lung ischemiareperfusion injury. Mol Med Rep.

[21] Liu YE, Tong CC, Zhang YB, Jin HX, Gao Y, Hou MX (2015). Effect of dexmedetomidine on rats with renal ischemia-reperfusion injury and the expression of tight junction protein in kidney. Int J Clin Exp Med.

[22] Sahin T, Begec Z, Toprak HI, Polat A, Vardi N, Yucel A, Durmus M, Ersoy MO (2013). The effects of dexmedetomidine on liver ischemia-reperfusion injury in rats. J Surg Res.

[23] Chen JH, Yu GF, Jin SY, Zhang WH, Lei DX, Zhou SL, Song XR (2015). Activation of alpha2 adrenoceptor attenuates lipopolysaccharide-induced hepatic injury. Int J Clin Exp Pathol.

[24] Klimscha W, Weinstabl C, Ilias W, Mayer N, Kashanipour A, Schneider B, Hammerle A (1993). Continuous spinal anesthesia with a microcatheter and low-dose bupivacaine decreases the hemodynamic effects of centroneuraxis blocks in elderly patients. Anesth Analg.

[25] Biboulet P, Jourdan A, Van Haevre V, Morau D, Bernard N, Bringuier S, Capdevila X. Hemodynamic profile of target-controlled spinal anesthesia compared with 2 target-controlled general anesthesia techniques in elderly patients with cardiac comorbidities. Reg Anesth Pain Med 2012;37(4):433–440. Epub 2012/05/23. 10.1097/AAP.0b013e318252e901 22609644

[26] Saumier N, Gentili M, Dupont H, Aubrun F (2013). Postoperative intravenous morphine titration in PACU after bariatric laparoscopic surgery. Ann Fr Anesth Reanim.

[27] Abou Hammoud H, Simon N, Urien S, Riou B, Lechat P, Aubrun F (2009). Intravenous morphine titration in immediate postoperative pain management: population kinetic-pharmacodynamic and logistic regression analysis. Pain.

[28] Aubrun F, Monsel S, Langeron O, Coriat P, Riou B (2001). Postoperative titration of intravenous morphine. Eur J Anaesthesiol.

[29] Riker RR, Picard JT, Fraser GL (1999). Prospective evaluation of the Sedation-Agitation Scale for adult critically ill patients. Crit Care Med.

[30] Lerman J (1996). Study design in clinical research: sample size estimation and power analysis. Can J Anaesth.

[31] Chan AK, Cheung CW, Chong YK (2010). Alpha-2 agonists in acute pain management. Expert Opin Pharmacother.

[32] Gurbet A, Basagan-Mogol E, Turker G, Ugun F, Kaya FN, Ozcan B (2006). Intraoperative infusion of dexmedetomidine reduces perioperative analgesic requirements. Can J Anaesth.

[33] Schnabel A, Meyer-Friessem CH, Reichl SU, Zahn PK, Pogatzki-Zahn EM (2013). Is intraoperative dexmedetomidine a new option for postoperative pain treatment? A meta-analysis of randomized controlled trials. Pain.

[34] Ulger F, Bozkurt A, Bilge SS, Ilkaya F, Dilek A, Bostanci MO, Ciftcioglu E, Guldogus F (2009). The antinociceptive effects of intravenous dexmedetomidine in colorectal distension-induced visceral pain in rats: the role of opioid receptors. Anesth Analg.

[35] Sudo RT, Calasans-Maia JA, Galdino SL, Lima MC, Zapata-Sudo G, Hernandes MZ, Pitta IR (2010). Interaction of morphine with a new alpha2-adrenoceptor agonist in mice. J Pain.

[36] Turgut N, Turkmen A, Ali A, Altan A (2009). Remifentanil-propofol vs dexmedetomidine-propofol--anesthesia for supratentorial craniotomy. Mid East J Anaesth.

[37] Hwang W, Lee J, Park J, Joo J (2015). Dexmedetomidine versus remifentanil in postoperative pain control after spinal surgery: a randomized controlled study. BMC Anesthesiol.

[38] Haselman MA (2008). Dexmedetomidine: a useful adjunct to consider in some high-risk situations. AANA J.

[39] Arain SR, Ruehlow RM, Uhrich TD, Ebert TJ (2004). The efficacy of dexmedetomidine versus morphine for postoperative analgesia after major inpatient surgery. Anesth Analg.

[40] Celerier E, Gonzalez JR, Maldonado R, Cabanero D, Puig MM (2006). Opioid-induced hyperalgesia in a murine model of postoperative pain: role of nitric oxide generated from the inducible nitric oxide synthase. Anesthesiology.

[41] Rivosecchi RM, Rice MJ, Smithburger PL, Buckley MS, Coons JC, Kane-Gill SL (2014). An evidence based systematic review of remifentanil associated opioid-induced hyperalgesia. Expert Opin Drug Saf.

[42] Kim YK, Kim CS, Chung GH, Han YM, Lee SY, Jin GY, Lee JM (2006). Radiofrequency ablation of hepatocellular carcinoma in patients with decompensated cirrhosis: evaluation of therapeutic efficacy and safety. AJR Am J Roentgenol.

[43] Cizginer S, Tatli S, Hurwitz S, Tuncali K (2011). vanSonnenberg E, Silverman SG. Biochemical and hematologic changes after percutaneous radiofrequency ablation of liver tumors: experience in 83 procedures. J Vasc Interven Radiol.

[44] Kucuk A, Yaylak F, Cavunt-Bayraktar A, Tosun M, Arslan M, Comu FM, Kavutcu M (2014). The protective effects of dexmedetomidine on hepatic ischemia reperfusion injury. Bratislavske Lekarske Listy.

[45] Wang ZX, Huang CY, Hua YP, Huang WQ, Deng LH, Liu KX (2014). Dexmedetomidine reduces intestinal and hepatic injury after hepatectomy with inflow occlusion under general anaesthesia: a randomized controlled trial. Br J Anaesth.

[46] Abdel Hamid AM, Abo Shady AF, Abdel Azeem ES (2010). Remifentanil infusion as a modality for opioid-based anaesthesia in paediatric practice. Ind J Anaesth.

[47] Scott LJ, Perry CM (2005). Remifentanil: a review of its use during the induction and maintenance of general anaesthesia. Drugs.

[48] Maruyama K, Nishikawa Y, Nakagawa H, Ariyama J, Kitamura A, Hayashida M (2010). Can intravenous atropine prevent bradycardia and hypotension during induction of total intravenous anesthesia with propofol and remifentanil?. J Anesth.

[49] Ciftci T, Erbatur S, Ak M (2015). Comparison of the effects of dexmedetomidine and remifentanil on potential extreme haemodynamic and respiratory response following mask ventilation and laryngoscopy in patients with mandibular fractures. Eur Rev Med Pharmacol Sci.

[50] Lee YY, Wong SM, Hung CT (2007). Dexmedetomidine infusion as a supplement to isoflurane anaesthesia for vitreoretinal surgery. Br J Anaesth.

[51] Menda F, Koner O, Sayin M, Ture H, Imer P, Aykac B (2010). Dexmedetomidine as an adjunct to anesthetic induction to attenuate hemodynamic response to endotracheal intubation in patients undergoing fast-track CABG. Ann Card Anaesth.

[52] Bloor BC, Ward DS, Belleville JP, Maze M (1992). Effects of intravenous dexmedetomidine in humans. II. Hemodynamic changes. Anesthesiology.

[53] Arcangeli A, D'Alo C, Gaspari R (2009). Dexmedetomidine use in general anaesthesia. Curr Drug Targets.

[54] Jessen Lundorf L, Korvenius Nedergaard H, Moller AM (2016). Perioperative dexmedetomidine for acute pain after abdominal surgery in adults. Cochrane Database Syst Rev.

[55] Basar H, Akpinar S, Doganci N, Buyukkocak U, Kaymak C, Sert O, Apan A (2008). The effects of preanesthetic, single-dose dexmedetomidine on induction, hemodynamic, and cardiovascular parameters. J Clin Anesth.

[56] Aho M, Lehtinen AM, Erkola O, Kallio A, Korttila K (1991). The effect of intravenously administered dexmedetomidine on perioperative hemodynamics and isoflurane requirements in patients undergoing abdominal hysterectomy. Anesthesiology.

[57] Keniya VM, Ladi S, Naphade R (2011). Dexmedetomidine attenuates sympathoadrenal response to tracheal intubation and reduces perioperative anaesthetic requirement. Ind J Anaesth.

[58] Harsoor SS, Rani DD, Lathashree S, Nethra SS, Sudheesh K (2014). Effect of intraoperative Dexmedetomidine infusion on Sevoflurane requirement and blood glucose levels during entropy-guided general anesthesia. J Anaesthesiol Clin Pharmacol.

[59] Anjum N, Tabish H, Debdas S, Bani HP, Rajat C, Anjana Basu GD (2015). Effects of dexmedetomidine and clonidine as propofol adjuvants on intra-operative hemodynamics and recovery profiles in patients undergoing laparoscopic cholecystectomy: A prospective randomized comparative study. Avicenna J Med.

[60] Patel CR, Engineer SR, Shah BJ, Madhu S (2012). Effect of intravenous infusion of dexmedetomidine on perioperative haemodynamic changes and postoperative recovery: A study with entropy analysis. Ind J Anaesth.

[61] Turan G, Ozgultekin A, Turan C, Dincer E, Yuksel G (2008). Advantageous effects of dexmedetomidine on haemodynamic and recovery responses during extubation for intracranial surgery. Eur J Anaesthesiol.

[62] Aksu R, Akin A, Bicer C, Esmaoglu A, Tosun Z, Boyaci A (2009). Comparison of the effects of dexmedetomidine versus fentanyl on airway reflexes and hemodynamic responses to tracheal extubation during rhinoplasty: A double-blind, randomized, controlled study. Curr Ther Res Clin Exp.

