# Supplementary material for: Comparison of dexmedetomidine vs. remifentanil combined with sevoflurane during radiofrequency ablation of hepatocellular carcinoma: a randomized controlled trial
Source: Trials. 2019 Jan 8;20:28. doi: 10.1186/s13063-018-3010-z (PMC6326039; doi:10.1186/s13063-018-3010-z)
Supplement: Supplementary file 1 — CONSORT-Equity checklist. (DOCX 18 kb) [file 13063_2018_3010_MOESM1_ESM.docx]

**Table 1: The standard CONSORT 2010 items to the left, with proposed CONSORT-Equity extensions to the right**

| **Section** | **Item** | **Standard CONSORT Item** | **Proposed Extension for Equity** | **Pg.** |
| --- | --- | --- | --- | --- |
| **Title** | | | | |
| **Title** | 1a | Identification as a randomised trial in the title | If health equity is a major focus, consider using the term “health equity” in the title. | 1 |
| **Abstract** | | | | |
| **Structured Summary** | 1b | Structured summary of trial design, methods, results, and conclusions (for specific guidance see CONSORT for abstracts) | State research question(s) related to health equity | 3-4 |
|  | 1c |  | Present results of all planned health equity analyses |  |
|  | 1d |  | Describe extent and limits of applicability to populations of interest across PROGRESS-Plus characteristics |  |
| **Introduction** | | | | |
| **Background** | 2a | Scientific background and explanation of rationale | Describe rationale for focus on health equity | 4-6 |
| **Objective** | 2b | Specific objectives or hypotheses | State the objective being addressed with reference to health equity | 6 |
| **Methods** | | | | |
| **Trial Design** | 3a | Description of trial design (such as parallel, factorial) including allocation ratio | Describe aspects of trial design that were chosen to answer equity questions | 6-8 |
|  | 3b | Important changes to methods after trial commencement (such as eligibility criteria), with reasons |  | 8-9 |
| **Participants** | 4a | Eligibility criteria for participants | Report population eligibility criteria across relevant PROGRESS-Plus characteristics. | 6-7 |
|  | 4b | Settings and locations where the data were collected | Report context and relationship to health inequity | 9-10 |
|  | 4c |  | Report details of partnerships with populations and communities, where applicable. |  |
| **Intervention** | 5 | The interventions for each group with sufficient details to allow replication, including how and when they were actually administered | Report whether comparator intervention is the standard of care, and whether it has equity implications. | 7-9 |
| **Outcomes** | 6a | Completely defined pre-specified primary and secondary outcome measures, including how and when they were assessed | Report whether outcomes were identified as relevant and important to population(s) across PROGRESS-Plus characteristics and how this was done | 10 |
|  | 6b | Any changes to trial outcomes after the trial commenced, with reasons |  | 8-10 |
| **Sample Size** | 7a | How sample size was determined | Report whether analyses focused on health equity objectives are powered to detect differences. | 10-11 |
|  | 7b | When applicable, explanation of any interim analyses and stopping guidelines |  | 8-9 |
| **Randomisation Sequence Generation** | 8a | Method used to generate the random allocation sequence |  | 7 |
|  | 8b | Type of randomisation; details of any restriction (such as blocking and block size) | Report whether randomisation was stratified on PROGRESS-Plus characteristic(s) | 7 |
| **Allocation Concealment Mechanism** | 9 | Mechanism used to implement the random allocation sequence (such as sequentially numbered containers), describing any steps taken to conceal the sequence until interventions were assigned |  | 7 |
| **Implementation** | 10 | Who generated the random allocation sequence, who enrolled participants, and who assigned participants to interventions |  | 7 |
| **Blinding** | 11a | If done, who was blinded after assignment to interventions (for example, participants, care providers, those assessing outcomes) and how |  | 7 |
|  | 11b | If relevant, description of the similarity of interventions |  | 7 |
| **Statistical Methods** | 12a | Statistical methods used to compare groups for primary and secondary outcomes |  | 10-11 |
|  | 12b | Methods for additional analyses, such as subgroup analyses and adjusted analyses | Report details of additional analyses focused on health equity, including whether analyses to estimate heterogeneity of effects between population subgroups were done on an additive or multiplicative scale, and whether pre-specified. | none |
| **Ethical Concerns** | a | New item** | Report details of ethical clearance and informed consent | 6 |
| **Results** | | | | |
| **Participant flow (a diagram is strongly recommended)** | 13a | For each group, the numbers of participants who were randomly assigned, received intended treatment, and were analyzed for the primary outcome | Describe for each group, numbers of participants who were assigned, received and who were analyzed across relevant PROGRESS-Plus characteristics | 11-12 |
|  | 13b | For each group, losses and exclusions after randomisation, together with reasons | Describe for each group, losses and exclusions after randomisation across relevant PROGRESS-Plus characteristics, with reasons. | 11-12 |
| **Recruitment** | 14a | Dates defining the periods of recruitment and follow-up | Report whether methods of recruitment were designed to reach populations across relevant PROGRESS-Plus characteristics. | 6-7 |
|  | 14b | Why the trial ended or was stopped |  | 8-9 |
| **Baseline Data** | 15 | A table showing baseline demographic and clinical characteristics for each group | Present the baseline characteristics also across relevant PROGRESS-Plus characteristics. | 11-12 |
| **Numbers Analyzed** | 16 | For each group, number of participants (denominator) included in each analysis and whether the analysis was by original assigned groups |  | 11-12 |
| **Outcomes and Estimation** | 17a | For each primary and secondary outcome, results for each group, and the estimated effect size and its precision (such as 95% confidence interval) |  | 12-13 |
|  | 17b | For binary outcomes, presentation of both absolute and relative effect sizes is recommended |  | 12-13 |
| **Ancillary Analysis** | 18a | Results of any other analyses performed, including subgroup analyses and adjusted analyses, distinguishing pre-specified from exploratory | Give the results of additional analytic approaches related to equity objectives distinguishing pre-specified from exploratory. | 12-13 |
|  | 18b |  | Details of implementation (coverage, intensity) in each trial arm across relevant PROGRESS-Plus characteristics |  |
| **Harms** | 19 | All important harms or unintended effects in each group (for specific guidance see CONSORT for harms ) | Report whether intervention generated inequities (e.g. unintended effects) were assessed | 12 |
| **Discussion** | | | | |
| **Limitation** | 20 | Trial limitations, addressing sources of potential bias, imprecision, and, if relevant, multiplicity of analyses | Report any limitations related to assessing effects on health equity. | 18 |
| **Generalizability** | 21 | Generalisability (external validity, applicability) of the trial findings | In addition, report applicability related to population of interest across PROGRESS-Plus characteristics. | 14 |
| **Interpretation** | 22 | Interpretation consistent with results, balancing benefits and harms, and considering other relevant evidence |  | 14-18 |
| **Other Information** | | | | |
| **Registration** | 23 | Registration number and name of trial registry |  | 4, 6 |
| **Protocol** | 24 | Where the full trial protocol can be accessed, if available |  | 6-10 |
| **Funding** | 25 | Sources of funding and other support (such as supply of drugs), role of funders |  | 19 |
