# Supplementary material for: Comparison of dexmedetomidine vs. remifentanil combined with sevoflurane during radiofrequency ablation of hepatocellular carcinoma: a randomized controlled trial
Source: Trials. 2019 Jan 8;20:28. doi: 10.1186/s13063-018-3010-z (PMC6326039; doi:10.1186/s13063-018-3010-z)
Supplement: Supplementary file 2 — CONSORT-Equity flow diagram. Intervention A denotes remifentanil and intervention B denotes dexmedetomidine. (DOC 70 kb) [file 13063_2018_3010_MOESM2_ESM.doc]

**
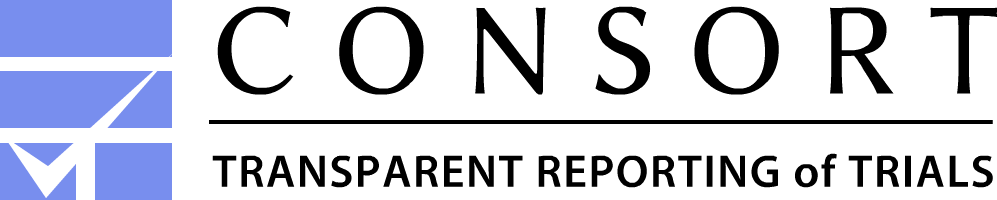
**

**CONSORT 2010 Flow Diagram[[1]](#footnote-2)**

Allocated to intervention B (n= 47 )

♦ Received allocated intervention (n= 47 )

♦ Did not receive allocated intervention (give reasons) (n= 0 )

Lost to follow-up (give reasons) (n= 0 )

Discontinued intervention (give reasons) (n=4)

Lost to follow-up (give reasons) (n= 0 )

Discontinued intervention (give reasons) (n=3)

**Male:** Allocated to intervention A (n= 44 )

 Received allocated intervention (n= 44 )

 Did not receive allocated intervention (give reasons) (n= 0 )

**Female:** Allocated to intervention (n= 3 )

♦ Received allocated intervention (n= 3 )

♦ Did not receive allocated intervention (give reasons) (n= 0 )

**Male:** Allocated to intervention B (n= 38 )

 Received allocated intervention (n= 38 )

 Did not receive allocated intervention (give reasons) (n= 0 )

**Female:** Allocated to intervention B (n= 9 )

♦ Received allocated intervention (n= 9 )

♦ Did not receive allocated intervention (give reasons) (n= 0 )

**Follow-Up**

Analysed (n= 47 )

♦ Excluded from analysis (give reasons) (n=0)

Analysed (n= 47 )

♦ Excluded from analysis (give reasons) (n=0)

**Analysis**

**Male:** Lost to follow-up (give reasons) (n= 0 )

Discontinued intervention (give reasons) (n= 4 )

2 significant bradycardia

2 significant hypotension

**Male:** Lost to follow-up (give reasons) (n= 0 )

Discontinued intervention (give reasons) (n= 3 )

1 significant bradycardia

2 significant hypotension

**Female:** Lost to follow-up (give reasons) (n= 0 )

Discontinued intervention (give reasons) (n= 0 )

**Female:** Lost to follow-up (give reasons) (n= 0 )

Discontinued intervention (give reasons) (n= 0 )

**Male:** Analysed (n= 44 )

♦ Excluded from analysis (give reasons) (n= 0 )

**Female:** Analysed (n= 3)

♦ Excluded from analysis (give reasons) (n= 0 )

**Male:** Analysed (n=38 )

♦ Excluded from analysis (give reasons) (n= 0 )

**Female:** Analysed (n=9 )

♦ Excluded from analysis (give reasons) (n= 0 )

Assessed for eligibility (n= 249 )

Excluded (n= 155 )

♦  Not meeting inclusion criteria (n=155)

♦  Declined to participate (n= 0 )

♦  Other reasons (n= 0 )

**Enrollment**

**Allocation**

Randomized (n= 94 )

Allocated to intervention A (n= 47)

**Analysis**

♦ Received allocated intervention (n= 47 )

♦ Did not receive allocated intervention (give reasons) (n= 0 )

Analysed (n= )
♦ Excluded from analysis (give reasons) (n= )

Analysed (n= )
♦ Excluded from analysis (give reasons) (n= )

Analysed (n= )
♦ Excluded from analysis (give reasons) (n= )

Analysed (n= )
♦ Excluded from analysis (give reasons) (n= )

Analysed (n= )
♦ Excluded from analysis (give reasons) (n= )

Analysed (n= )
♦ Excluded from analysis (give reasons) (n= )

**Analysis**

1. Sex used as an example (male/female) but other PROGRESS characteristics could be used [↑](#footnote-ref-2)
